# A Comprehensive Worksite Wellness Program in Austin, Texas: Partnership Between Steps to a Healthier Austin and Capital Metropolitan Transportation Authority

**Published:** 2009-03-15

**Authors:** Lynn Davis, Karina Loyo, Rick Schwertfeger, Aerie Glowka, Lisa Danielson, Cecily Brea, Alyssa Easton, Shannon Griffin-Blake

**Affiliations:** City of Austin/Travis County Health and Human Services; City of Austin/Travis County Health and Human Services Department, Austin, Texas; City of Austin/Travis County Health and Human Services Department, Austin, Texas; Health & Lifestyles Corporate Wellness, Inc, Austin, Texas; Health & Lifestyles Corporate Wellness, Inc, Austin, Texas; Health & Lifestyles Corporate Wellness, Inc, Austin, Texas; CDC’s Healthy Communities Program, Atlanta, Georgia; CDC’s Healthy Communities Program, Atlanta, Georgia

## Abstract

**Background:**

In 2003, Steps to a Healthier Austin was funded by the Centers for Disease Control and Prevention to implement chronic disease prevention and health promotion activities. We report Steps to a Healthier Austin's partnership with Health & Lifestyles Corporate Wellness, Inc (Health & Lifestyles), to provide a worksite wellness program for Capital Metropolitan Transportation Authority (Capital Metro), Austin's local transit authority.

**Context:**

Capital Metro employs 1,282 people. In 2003, Health & Lifestyles was hired to help promote healthier lifestyles, increase employee morale, and combat rising health care costs and absenteeism rates.

**Methods:**

Health & Lifestyles provided consultations with wellness coaches and personal trainers, a 24-hour company fitness center, personalized health assessments, and preventive screenings. The program expanded to include healthier food options, cash incentives, health newsletters, workshops, dietary counseling, smoking cessation programs, and a second fitness center.

**Consequences:**

Participants in the wellness program reported improvements in physical activity, healthy food consumption, weight loss, and blood pressure. Capital Metro's total health care costs increased by progressively smaller rates from 2003 to 2006 and then decreased from 2006 to 2007. Absenteeism has decreased by approximately 25% since the implementation of the program, and the overall return on the investment was calculated to be 2.43.

**Interpretation:**

Since the implementation of the wellness program in 2003, Capital Metro has seen a reduction in costs associated with employee health care and absenteeism.

## Background

Capital Metropolitan Transportation Authority (Capital Metro) provides public transportation services to the city of Austin, Texas. This organization, established in 1985, has 1,282 permanent employees and 250 contract employees. Approximately half (668) of the permanent employees are bus operators. The organization, with a small workforce, serves a sizeable number of residents (approximately 738,000) in the growing city of Austin (1).

As with many American companies, health care costs reached record levels for Capital Metro in 2003 — almost $10.6 million. Before 2003, Capital Metro's group insurance premiums increased by 40% or more annually (V. Keeling, written communication, October 2008). In 2003, Capital Metro became a self-insured company. By becoming self-insured, Capital Metro was able not only to know exactly what it spent annually on health insurance but also to gain more control over these expenses. From 2003 to 2004, health care costs increased to $13.4 million (a 26.4% increase). Health care costs at Capital Metro are higher for certain people. In 2007, the average medical plan cost for employees with diabetes, hypertension, or high cholesterol was $3,454 per month. Essentially, an employee with a chronic disease incurred expenses 2 to 3 times higher than an employee without a chronic disease.

Absenteeism also greatly increased during the early 2000s. Absenteeism costs in 2001 were almost $2 million, and by 2004 they had risen to almost $2.3 million — an increase of almost 15% in only 3 years — during which time the size of the workforce remained fairly static (1). Unscheduled absenteeism requires companies to hire last-minute temporary staff and to pay additional overtime, which contributes to a higher overall labor cost. Because of the rapidly increasing costs of health care and absenteeism, part of Capital Metro's response was to begin an employee wellness program in 2003.

## Context

Capital Metro delivers its wellness programs through Health & Lifestyles Corporate Wellness, Inc (Health & Lifestyles), a private company based in Austin. This company provides comprehensive worksite wellness solutions for employers by using evidence-based programs and practices. Health & Lifestyles created and has been managing the Capital Metro worksite wellness program since its inception in January 2003.

Steps to a Healthier Austin is a community-based program, funded by a cooperative agreement from the Centers for Disease Control and Prevention, designed to reduce diabetes, asthma, and obesity by improving nutrition, increasing physical activity, and decreasing tobacco use. The Steps to a Healthier Austin intervention area includes 20 contiguous zip codes that encompass the eastern half of the city of Austin and Travis County. This area has a population of 460,041, of which 41% are Hispanic and 14% are African American (2). In 2003, the median household income in the intervention area was $37,424, approximately 40% lower than in other parts of Travis County, and the death rate was 22% higher (3).

The intervention area population also has higher rates of chronic disease compared with the rest of Travis County. An estimated 12% of adults have been diagnosed with asthma, 7% have been diagnosed with diabetes, 55% are overweight, and 26% are obese. Overall, 21% of the adult Steps to a Healthier Austin population smoke cigarettes, 26% report no leisure-time physical activity, and only 25% meet the minimum recommended daily consumption of 5 servings of fruits and vegetables (unpublished Steps to a Healthier Austin Behavioral Risk Factor Surveillance System data). In addition, racial/ethnic health disparities are documented in the intervention area; African Americans have higher death rates from heart disease, hypertension, cerebrovascular disease, and diabetes than do whites. Both Hispanics and African Americans in the intervention area are 2.5 times more likely than whites to die from diabetes (3).

To address these issues, Steps to a Healthier Austin partnered with key organizations in the intervention area. Capital Metro was invited to be Steps to a Healthier Austin's worksite wellness partner for the Steps to a Healthier Austin consortium. Capital Metro was chosen for several reasons. First, Capital Metro's workforce is ethnically diverse (33.5% African American and 27.7% Hispanic). Second, Capital Metro's headquarters is in the intervention area, where approximately 47% of Capital Metro employees live (V. Keeling, oral communication, January 2009). Third, Capital Metro had a promising worksite wellness program and was willing to be a part of the greater Steps to a Healthier Austin program. Finally, Capital Metro's workforce had high rates of chronic disease. In 2005, as part of the partnership between Capital Metro and Steps to a Healthier Austin, Capital Metro received an influx of Steps to a Healthier Austin funds to be used to enhance its wellness program. This increased financial commitment rounded out the offerings of the program and could have been responsible for the improvements in health care cost and absenteeism seen after 2005.

## Methods

Health & Lifestyles launched the Capital Metro wellness program in January 2003. Participants included bus operators, mechanics, and administrative employees. The initial program consisted of 1 full-time employee who was available to provide health information and one-on-one consulting and education, who made brochures on health information available, and who coordinated wellness seminars and demonstrations by local vendors. Over time, the wellness program expanded to include multiple offerings, including on-site fitness centers, healthier food options, cash incentives, health newsletters, workshops, dietary counseling, and smoking cessation programs. In 2005, Steps to a Healthier Austin made Health & Lifestyles, via Capital Metro, a formal consortium partner and since that time has funded the purchase of more equipment and the hiring of 1 part-time personal fitness trainer.

The first company fitness center was launched in June 2006 and is open 24 hours per day. The fitness center includes stationary weight lifting equipment, free weights, exercise bands, balls, exercise mats, shower facilities, and lockers. The fitness center is located at the corporate headquarters, and membership is only $5 per month for employees. Free assessments and personal training are provided Monday through Friday from 6:00 am to 6:00 pm.

Shortly after the new fitness center opened, Steps to a Healthier Austin funded the purchase of a TriFIT machine (Polar USA, Lake Success, New York). The machine provides employees with a full body assessment and plan for achieving fitness goals. A full body assessment includes weight, flexibility, body composition, maximal oxygen consumption (VO_2 max_), and other factors. The machine tracks employees' progress and provides data needed to determine their eligibility for cash incentives. It also provides a nutrition and exercise plan, to which the personal trainer and dietitian can refer when assisting each employee with his or her fitness goals. The TriFIT has been an integral part of the fitness component of Capital Metro's worksite wellness program, and Capital Metro recently purchased a second machine to meet the needs of the increasing number of employees participating in this program.

Cash incentives have also been a key part of the success of the employee wellness program. Cash incentives are provided if any of the following occurs:

- Blood pressure drops to 120/80 mm Hg or lower.
- Fasting plasma glucose drops to below 100 mg/dL (employees without diabetes) or 126 mg/dL (employees with diabetes).
- Total cholesterol drops to below 200 mg/dL.
- Body fat decreases by 3% or more in 6 months.
- The employee loses 10% of his initial weight.
- VO_2 max_ increases by 5 mL/kg/min in 6 months.

Cash incentives are also used to reward employees for joining weight-loss programs, for sustaining use of the on-site gym, and for achieving other health-related goals, such as participation in races and stopping smoking.

In addition to offering physical activity opportunities, as part of its wellness program, Capital Metro has improved access to healthy food. Capital Metro now provides what it calls its Healthy Options Cafe. Employees are given weekly discount coupons to purchase healthy cafeteria items; $5 worth of coupons are available each week for each Capital Metro employee and contractor. Capital Metro also instituted a healthy vending contract and stipulated that 60% of offerings had to be healthy choices, and healthy options are cheaper than less healthy options. No snacks can be "value sized," and all snacks must contain less than 5 g fat (nuts and seeds exempt) and less than 30 g carbohydrate (fruit exempt). Beverage choices are 25% water or sports drinks, 25% fruit juices, 25% low-calorie soft drinks, and 25% regular soft drinks.

Besides improving physical activity access and providing healthier food choices, Capital Metro offers on-site Weight Watchers classes. Employees are reimbursed up to $180 for the cost of the program if they lose 10% of their initial weight and if they attend 85% of meetings during a specified time period. In addition, employees earn $75 for losing 10% of their initial body weight, $125 for reaching their goal weight, $200 for maintaining their goal weight for 6 months, and an additional $200 each year the weight is maintained for up to 5 years.

Capital Metro also has a strong tobacco-cessation component in its wellness program. Employees receive up to $100 in reimbursement for over-the-counter nicotine replacement products, and they can earn $250 for every year they remain tobacco-free for up to 5 years.

Other elements of the wellness program include a wellness calendar and newsletter. The monthly calendar details health-related events and highlights health topics related to the national health calendar, with an emphasis on disease prevention. Additional promotions (educational programs, awareness campaigns, newsletters) are planned throughout the year relevant to diseases that are prevalent among the employee population.

Health promotion events offered on-site include health screenings, educational programs, cooking demonstrations with the Sustainable Food Center (Steps to a Healthier Austin's partner), chair massages, and stretching sessions. Employee health screenings are held throughout the year and include ultrasound stroke screening, vision and hearing screenings, mammograms, and glucose, cholesterol, and blood pressure testing. Screenings are offered at no charge.

Once each year, Capital Metro hosts a Health and Wellness Expo that presents information from vendors and conducts games with prizes. Among other activities, this event promotes the annual Texas Round-Up and invites employees and their families to participate in the family mile, 5K, or 10K event. Other programs offered throughout the year include healthy eating workshops presented by dietitians, a bike loan program, a walking club, and stress-management workshops. In 2008, a second fitness center opened at a different site.

## Consequences

Participation in the wellness program increased markedly from 2006 to 2007. Currently, 300 employees — approximately 25% of the workforce — are enrolled in the program. Of these, half are bus operators and the rest are administrative or managerial staff. The number of visits to the wellness center increased from 1,571 in 2006 to 3,174 in 2007. The number of fitness center workouts recorded increased from 2,137 in 2006 to 11,176 in 2007. The number of personal training sessions also increased, from 561 visits in 2006 to 2,348 visits in 2007.

Cash incentives have been received by 172 participants for achieving their wellness goals. Specifically, Capital Metro has paid $7,800 in weight loss incentives, $7,500 for smoking cessation, $10,200 for gym memberships, $4,500 for TriFIT assessments, and $1,500 for 5K and 10K participation. Total cash incentives paid to date have reached $31,500.

Eighty-six of 133 people who had at least 2 TriFIT assessments in 2007 lost weight, a total of 791 pounds or approximately 10 pounds per person and an average loss of 3% body fat. Participants' average blood pressure decreased by 4 mm Hg, but because of the fluctuating nature of blood pressure, we cannot necessarily consider this to be a significant decrease.

People participating in the TriFIT assessment can also complete an optional health risk assessment. Unfortunately, many people feel the assessment involves disclosing too much private information, and they do not find it to be a useful tool they should fill out multiple times. The repeat risk assessment data are limited, and no conclusions can be drawn from them at this time. Consequently, the program focuses on goal-oriented programming. Participants receive individual coaching and information to help them achieve individual goals, such as weight loss, increased fitness, and increased flexibility.

**Figure 1.**
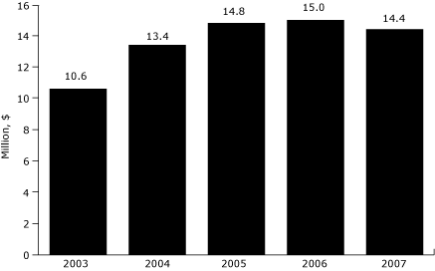
Total health care costs incurred by Capital Metropolitan Transportation Authority in Austin, Texas, 2003-2007.

**Figure 2.**
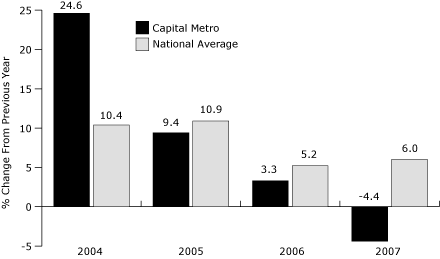
Change in health care costs incurred by Capital Metropolitan Transportation Authority (Capital Metro) in Austin, Texas, compared with the United States as a whole, 2003-2006.

From 2003, when the wellness program was launched, to 2006, Capital Metro's health care costs actually increased each year (Figure 1), but the magnitude of the change decreased every year. This trend must be viewed in the context of modest participation in early years of the program and increasing health care costs in the United States overall during this period (Figure 2). In 2007, when participation in the program increased dramatically, Capital Metro saw a 4% decrease in total health care costs. Similarly, rates of absenteeism among bus drivers remained stable at approximately 10% from 2001 through 2005. Rates declined to 8.2% in 2006 and declined again to 7.6% in 2007, which represented a total savings of $450,000 compared with the cost of the 10.1% absenteeism rate in 2004. Although we cannot say for certain that these decreases were attributable to wellness program participation, the reduction in health care costs and absenteeism from 2006 to 2007 suggests that a portion of participants were at risk for chronic diseases and that the program improved their health.

**Table:** 

**Table:** 

A return on investment (ROI) was calculated to show the effect of the program and to help management understand the benefits it provided. To calculate ROI, the health care and absenteeism savings were divided by the cost of the program during the 4 years ($750,000), for an ROI of 1.86 for health care savings, 0.57 in savings due to reduced absenteeism, and a total ROI of 2.43 from 2004 through 2007 (in other words, for every $1 that was spent on the program, $2.43 was saved).

## Interpretation

Since its beginning in 2003, the wellness program at Capital Metro has shown promising results in improving employee health and reducing costs associated with health care and absenteeism, and the financial benefits outweigh the annual investment (2.43 ROI). Employees engage in more physical activity, have better knowledge of disease management (diabetes and asthma), have better eating habits, and smoke less than they did before the program was implemented. Health care and absenteeism costs have been reduced and are continuing to decline, most likely as a result of the program. Managerial staff have reported that employee morale has increased since the program was implemented. Most importantly, however, we believe that the wellness program has the potential to reduce the prevalence and severity of chronic diseases, allowing Capital Metro employees to lead longer, healthier lives.
